# TRPV2-spike protein interaction mediates the entry of SARS-CoV-2 into macrophages in febrile conditions

**DOI:** 10.7150/thno.58781

**Published:** 2021-05-25

**Authors:** Jinrui Xu, Yuquan Yang, Zhaoyuan Hou, Hao Jia, Yujiong Wang

**Affiliations:** 1 Key Laboratory of the Ministry of Education for Conservation and Utilization of Special Biological Resources in the Western, Yinchuan 750021, China.; 2 College of Life Science, Ningxia University, Yinchuan 750021, Ningxia, China.; 3 Faculty of Basic Medicine, Shanghai Jiao tong University School of Medicine, Shanghai, China.

**Keywords:** SARS-CoV-2, Spike protein receptor-binding domain, TRPV2, SKF-96365, primary bovine alveolar macrophage

## Abstract

Severe acute respiratory syndrome coronavirus-2 (SARS-CoV-2) is a novel strain of highly contagious coronaviruses that infects humans. Prolonged fever, particularly that above 39.5 °C, is associated with SARS-CoV-2 infection. However, little is known about the pathological effects of fever caused by SARS-CoV-2.

**Methods:** Primary bovine alveolar macrophages (PBAMs), RAW264.7 mouse macrophages, and THP-1 human cells were transfected with plasmids carrying the genes encoding the SARS-CoV-2 spike (S) protein or receptor-binding domain (RBD). Proteins in the macrophages interacting with S-RBD at 39.5 °C or 37 °C were identified by immunoprecipitation-mass spectrometry. Glutathione *S*-transferase pulldown, surface plasmon resonance, and immunofluorescence were performed to evaluate the transient receptor potential vanilloid 2 (TRPV2) interaction with SARS-CoV-2-S-RBD at 39.5 °C. Using an RNA sequencing-based approach, cytokine gene expression induced by SARS-CoV-2 S transfection at 39.5 °C and 37.5 °C in primary alveolar macrophages was measured. Fluo-4 staining and enzyme-linked immunosorbent assays were used to assess the regulatory function of TRPV2 in intracellular Ca ^2+^ and cytokines under SARS-CoV-2-S-RBD at 39.5 °C. Additionally, cytokine release was examined after TRPV2 knockdown with shRNA oligonucleotides or inhibition using the SKF-96365 antagonist.

**Results:** We identified an interaction between the primary alveolar macrophage receptor TRPV2 and S-RBD under febrile conditions. Febrile temperature promotes Ca^2+^ influx through SARS-CoV-2 infection in PBAMs, further activates the NF-κB p65 signaling pathway, and enhances the secretion of cytokines. Furthermore, knockdown or antagonist (with SKF-96365) of TRPV2 significantly decreased the release of cytokines that drive the inflammatory response.

**Conclusion:** Collectively, our findings identified TRPV2 as a receptor of SARS-CoV-2 in conditions of febrile temperature, providing insight into critical interactions of SARS-CoV-2 with macrophages, as well as a useful resource and potential drug target for coronavirus disease 2019.

## Introduction

Coronavirus disease 2019 (COVID-19) is a highly transmittable viral infection caused by severe acute respiratory syndrome coronavirus-2 (SARS-CoV-2). Studies 1, 2 have shown that similarly to SARS-CoV, SARS-CoV-2 can enter cells via recognition and binding between its spike (S) protein and the angiotensin-converting enzyme 2 (ACE2) receptor on the surface membrane of cells to infect humans. However, the precise intermediate source of origin and its transfer mechanism into humans remain unknown. Infections with CoVs have been reported in several other animal species, such as cattle, horses, and swines 3. Similar to SARS-CoV-2, bovine coronavirus (BCoV) is a β-coronavirus 4. BCoV has a broad host range, including wild ruminants, humans, and livestock, and produces similar pathogenic symptoms in the respiratory and gastrointestinal tracts 4. These characteristics of BCoV suggest a connection between BCoV and SARS-CoV. However, the mechanism of SARS-CoV-2 infection in bovines is unclear.

Transient receptor potential vanilloid 2 (TRPV2) was originally isolated as a molecule sensitive to temperatures above 52 °C 5. TRPV2 plays an important role in innate immunity 6 and is mainly distributed in the endoplasmic reticulum in the absence of stimulation. Under certain stimulatory conditions, such as stimulation by chemokines, growth factors, or stress conditions, TRPV2 can be transferred from the endoplasmic reticulum to the cell membrane to promote Ca^2+^ entry into the cell and accumulation in pseudopodia, which can promote the migration of macrophages towards an inflamed area and promote the phagocytic function of macrophages 6. Inhibition of TRPV2 expression can block ligand-mediated macrophage migration and slow the stress-mediated inflammatory response 7.

In this study, we examined the role of fever in host innate immunity and found that febrile temperature promoted cytokine storms through SARS-CoV-2 S protein-mediated infection of primary bovine alveolar macrophages (PBAMs). Next, we performed immunoprecipitation (IP) in PBAMs, and TRPV2 was identified as a specific coeluted protein by mass spectrometry (MS). Interestingly, febrile temperature promotes Ca^2+^ influx through SARS-CoV-2 infection in PBAMs. Knockdown or inhibition of TRPV2 significantly decreased the release of cytokines and chemokines, which are factors that drive the inflammatory response in macrophages. Collectively, our findings identified TRPV2 as a novel receptor of SARS-CoV-2 infection under febrile conditions, providing insight into the critical interactions of SARS-CoV-2 with macrophages, as well as a useful resource and potential drug target for COVID-19 treatment.

## Methods

### PBAM isolation and culture

PBAMs were isolated as previously described 8 and were obtained from the lungs of 1-2-year-old Simendal cattle bred in a tuberculosis-free herd. The entire lung was removed post-mortem under sterile conditions with a portion of the trachea, and was intratracheally infused with 500 mL of D-Hank's solution (Biotopped, Beijing, China) containing 50 μg/mL of gentamicin, 2.5 μg/mL of amphotericin, and 100 μg/mL of penicillin and streptomycin (HyClone, Logan, UT, USA). The recovered bronchoalveolar lavage fluid was filtered by passing a 70 μm-pore nylon cell strainer prior to collection into sterile beakers and centrifugation at 1000 rpm for 10 min. The cell pellet was then resuspended in 20 mL RPMI-1640 medium (HyClone) supplemented with 10% fetal bovine serum (Gibco, Grand Island, NY, USA), 100 U/mL of penicillin, and 100 U/mL of streptomycin.

RAW264.7 mouse macrophages and THP-1 human cells were cultured in RPMI-1640 medium supplemented with 10% fetal bovine serum. This study was approved by the institutional review board of Ningxia University.

### Antibody, chemical, plasmid, and protein preparation

Antibodies were obtained commercially: anti - ACE2 (rabbit, ab87436, Abcam, Cambridge, UK), anti - ACE2 (mouse, YT0077, ImmunoWay Biotechnology, Plano, TX, USA), anti - TRPV2 (rabbit, YN3347, ImmunoWay Biotechnology), anti - HA (mouse, MMS-101P, Covance, Princeton, NJ, USA), anti - Flag (mouse, F3165, Sigma, St. Louis, MO, USA), anti - Flag (rabbit, F7425, Sigma), anti - Myc (mouse, 13-2500, Invitrogen, Carlsbad, CA, USA), anti - GAPDH (mouse, 60004-1-Ig, Proteintech, Rosemont, IL, USA), normal IgG (rabbit, sc-2027, Santa Cruz Biotechnology, Dallas, TX, USA), and glutathione beads (GE Healthcare, Little Chalfont, UK). Plasmids containing the viral spike (S) region or receptor-binding domain (RBD) of spike (His - SARS-CoV-2-S, His - SARS-CoV-2-S-RBD, respectively) were previously described 9. The Ca ^2+^ entry inhibitor SKF-96365 hydrochloride was purchased from MedChemExpress (Monmouth Junction, NJ, USA). Fluo 4-AM was purchased from Abcam (ab228555, Abcam, USA). Plasmids of HA-SARS-CoV-2-S and HA-SARS-CoV-2-S-RBD were constructed in our lab. Recombinant proteins were expressed in *Escherichia coli* BL21 cells grown in 200 mL Luria-Bertani medium to an A_600_ of 0.6 at 37 °C. Protein expression was induced by addition of 0.2 mM isopropyl-β-D-thiogalactoside before the cells were incubated overnight at 16 °C. To purify the His - tagged proteins, the cell pellets were resuspended in lysis buffer containing 50 mM Tris-HCl (pH 8.0), 500 mM NaCl, and 20 mM imidazole (pH 8.0) and then lysed with a high-pressure cell cracker. The cell lysates were centrifuged at 12,000 × rpm for 20 min at 4 °C. The supernatants were purified with Ni^2+^ Sepharose beads, washed with lysis buffer, and eluted with buffer containing 50 mM Tris-HCl (pH 8.0), 500 mM NaCl, and 300 mM imidazole (pH 8.0).

### IP and MS assays

The detailed methods for the IP assay and western blotting have been described previously 10. The protein of interest was enriched with anti - His and anti - TRPV2 (ImmunoWay, YN3347) antibodies and resolved by 8% SDS-PAGE. Silver staining was performed using a silver staining kit (Beyotime, Shanghai, China) according to the manufacturer's instructions, and binding proteins were detected by MS (Novogene Bioinformatics Technology Co., Ltd., Beijing, China).

### shRNA knockdown, transfection and retroviral infection

Short hairpin RNA (shRNA) oligonucleotides (Gene Pharma, Shanghai, China) were transfected using Lipofectamine LTX. Supernatants containing viruses were packed from 293T cells. When growing to 60 - 80% confluency, cells were infected with viral supernatants, and 5 μg/mL puromycin was added to select the stable cells as mass pools. ShRNA oligonucleotides against TRPV2 had the following sequences:

- shRNA1: 5' - GGAAGACCUGUCUGAUGAATT- 3';
- shRNA2: 5' - GCUUCCAGUACACGGGUAUTT- 3', and;
- shRNA3: 5' - GCUCUUGGCCUAUGUGCUUTT- 3'.

The shRNA oligonucleotides against bovine ACE-2 had the following sequences:

- shRNA1: 5' - GGAGGUGGAUGGUCUUUAATT- 3';
- shRNA2: 5' - GCAGAGAUUAAACCAUUAUTT- 3'.

The shRNA oligonucleotides against bovine neuropilin 1 had the following sequences: shRNA1, 5' - GGGAGAACAAGGUGUUUAUTT- 3'; and shRNA2, 5' - CAGGGCCAUUUCUCUUUAUTT- 3'.

### Measurement of Intracellular Ca^2+^

PBAMs were cultured *in vitro* under His - S-RBD protein (0.5 μg/mL) at 37 °C or 39.5 °C for 24 h, and cells were washed twice with Hank's balanced salt solution and incubated in a final concentration of 5 μM Fluo 4-AM (Abcam) for 30 min at room temperature. After two washes with pre-warmed Hank's balanced salt solution, the cells were further incubated for 30 min at 37 °C or 39.5 °C, and images were acquired with a Zeiss Axioskop 40 microscope (Carl Zeiss, Oberkochen, Germany) with the following settings: excitation laser = 405 nm and emission gate center = 519 nm. The mean fluorescence intensity of Ca ^2+^ was measured with Image Pro Plus 6.0 software (Media Cybernetics, Rockville, MD, USA). Five fields/well and approximately 15 - 20 cells per field were examined in each group (n = 6).

### Indirect immunofluorescence staining (IF)

RAW264.7 cells were cultured *in vitro* under His - S-RBD (0.5 μg/mL) and His - Vector protein (0.5 μg/mL) at 37 °C or 39.5 °C for 24 h, and the cells were washed with PBS and fixed in 500 mL 4% paraformaldehyde for 20 min at room temperature (RT). The cells were washed three times with PBS for 5 min each, permeabilized in 2% Triton X-100 in PBS for 20 min at RT, and blocked with buffer (2% bovine serum albumin in PBS) for 30 min with gentle shaking at RT. The cells were then incubated in blocking buffer containing diluted primary antibodies (anti - TRPV2, anti - SARS-CoV2-S, anti - His) overnight at 4 °C and washed three times with PBS for 5 min each time. The cells were incubated with secondary antibodies in blocking buffer for 1 h at RT in the dark, and then washed three times with PBS for 5 min each time. The cells were incubated with DAPI (0.1 µg/mL) for 10 min, washed three times with PBS for 5 min, and fixed with 75% ethanol. Images were acquired using a confocal microscope.

### Bovine cytokine array

A bovine cytokine array (RayBiotech, Peachtree Corners, GA, USA) was used to screen for differential expression of more than 30 proteins, including cytokines, chemokines, inflammatory mediators, growth factors, and matrix metalloproteinases (H-Wayen Biotechnologies, Shanghai, China). Following plasmid transfection and incubation, the cells were cultured in fetal bovine serum-free medium for an additional 24 h. The supernatants were collected by centrifugation at 2,000 ×*g* for 10 min and were tested according to the Raybiotech protocol.

### Statistical analysis

Data shown as the mean ± standard deviation (SD) were analyzed by independent Student's *t*-test. Statistical analysis was performed using SPSS 18.0 software (SPSS, Inc., Chicago, IL, USA).

## Results

### TRPV2 interacts with SARS-CoV-2-S-RBD at 39.5 °C in macrophages and THP-1

**Figure 1:**
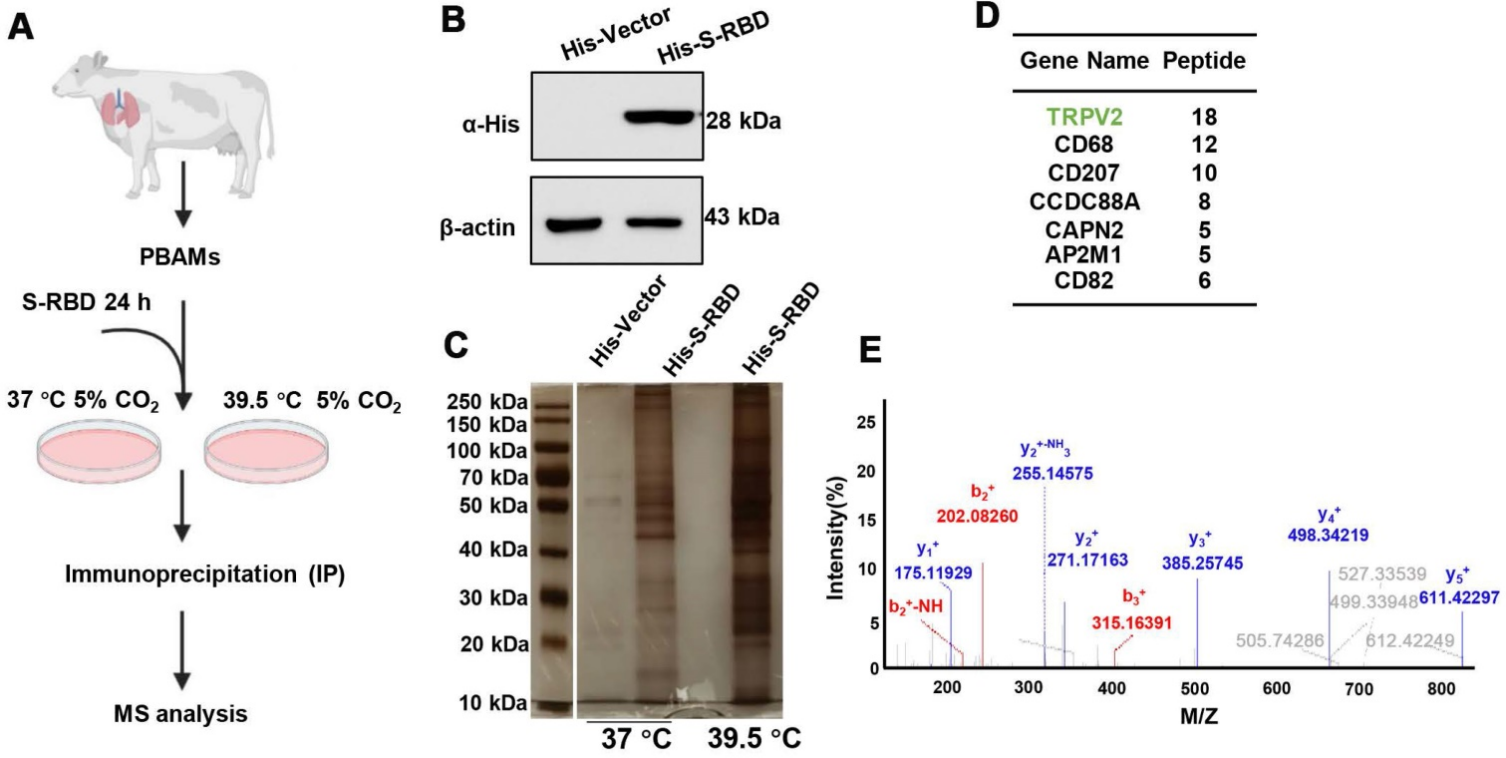
** Experimental design to identify proteins binding to SARS-CoV-2 spike and receptor binding protein at 39.5 °C in PBAMs. (A)** Schematic of the immunoprecipitation- mass spectrometry (IP-MS) experiment for identifying proteins associated with His - SARS-CoV2-S-RBD at 39.5 °C. **(B)** Western blot analysis of purified His - SARS-CoV-2 S-RBD protein using a polyclonal antibody against α-His antibody by SDS-PAGE. **(C)** Silver staining image showing His - SARS-CoV-2 S-RBD-interacting proteins in PBAMs under 37 °C and 39.5 °C. **(D)** MS analysis of S-RBD-associated proteins. Proteins with highest score of unique peptides are listed. **(E)** Tandem mass spectrometry analysis identifying a peptide derived from TRPV2.

**Figure 2:**
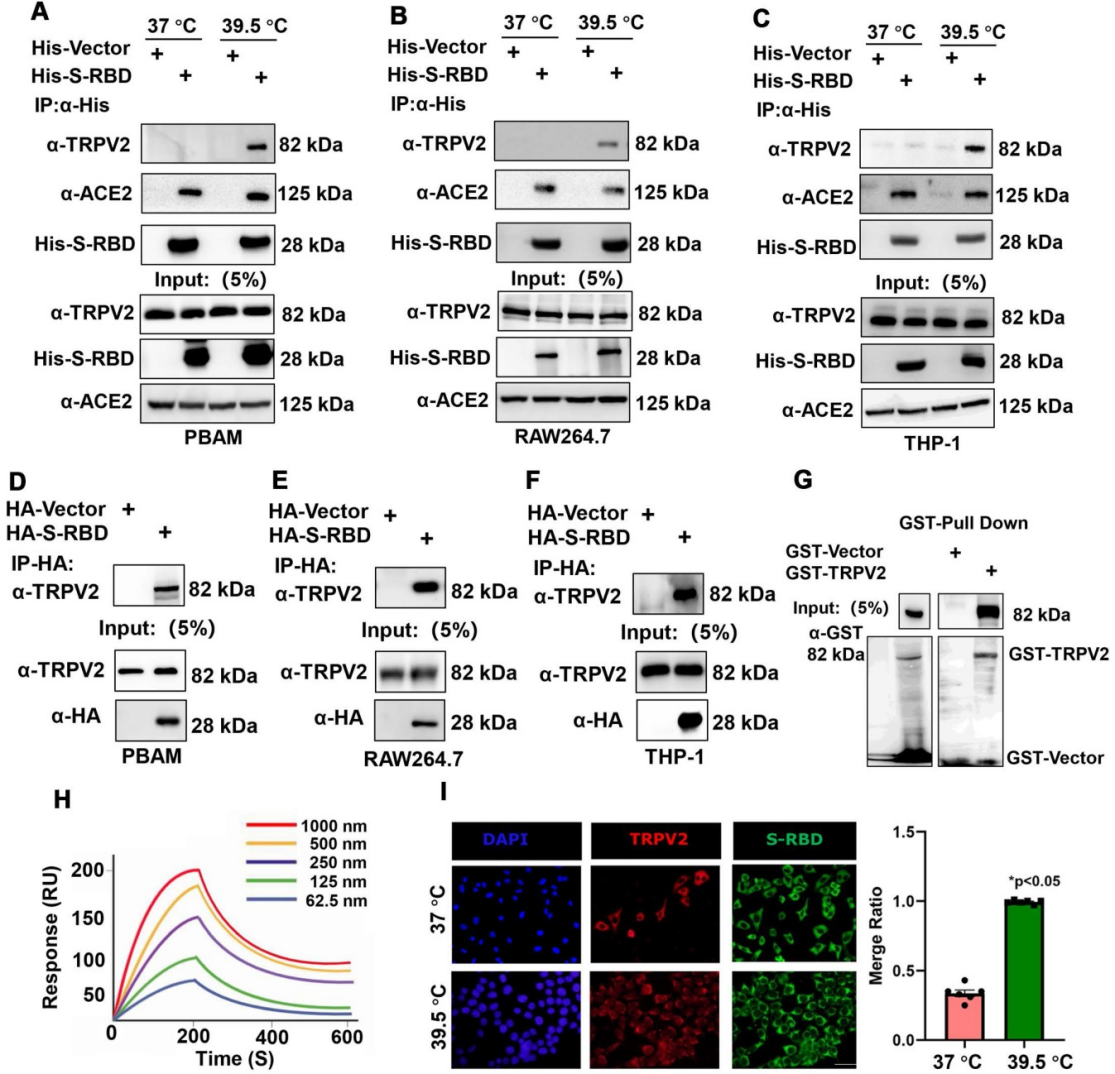
** SARS-CoV-2 S-RBD directly interacts with TRPV2. (A-C)** SARS-CoV-2 S-RBD interaction with endogenous TRPV2 at 39.5 ℃ in PBAMs (A), RAW264.7 cells (B), and THP-1 cells (C). **(D-F)** HA - S-RBD and HA - Vector were co-expressed in PBAMs (D), RAW264.7 cells (E), and THP-1 cells (F) under 39.5 ℃, and co-IP assays showed that TRPV2 interacted with S-RBD. **(G)** Interaction of TRPV2 and S-RBD was detected by GST-pulldown assay. **(H)** Interaction of TRPV2 and S-RBD was detected by surface plasmon resonance assay. Data are shown as the mean ± SD (n = 3). **(I)** Left: IF performed with primary anti - S-RBD and anti - TRPV2 antibodies and secondary antibodies (488 nm, donkey anti-mouse and 568 nm, donkey anti - rabbit). Scale bars represent 20 µm. Right: Ratio of TRPV2 to S-RBD. The ratio was calculated using the mean fluorescence intensity using Image J software from TRPV2 to S-RBD immunofluorescence data.

To understand the role of fever induced by SARS-CoV-2 infection in the macrophage response, PBAMs were cultured *in vitro* in the presence of His - S-RBD protein (0.5 μg/mL) at 37 °C or 39.5 °C for 24 h (Figure 1A, B). S-RBD-interacting proteins were subjected to affinity purification at 37 °C or 39.5 °C by IP-MS with an α-HA antibody in PBAMs. The purified protein complexes were resolved by SDS-PAGE and visualized by silver staining, and compared with 37 °C, new bands were observed under 39.5 °C (Figure 1C). The bands were then isolated and analyzed by MS, and a large number of proteins were identified, among which TRPV2 showed the highest unique peptide score (Figure 1D, E). We further examined the interaction between TRPV2 and S-RBD by performing co-IP assays in PBAMs at 37 °C or 39.5 °C. Co-IP analysis confirmed the interaction between TRPV2 and S-RBD at 39.5 °C but not at 37 °C (Figure 2A). Furthermore, we examined the interaction of TRPV2 and S-RBD at 39.5 °C in human THP-1 cells and mouse macrophages (RAW264.7) (Figure 2B, C). Next, we co-expressed HA-S-RBD and HA-Vector in PBAM at 39.5 °C, the co-IP assays showed that TRPV2 interacted with S-RBD (Figure 2D). Furthermore, we examined the interaction of TRPV2 and S-RBD at 39.5 °C in THP-1 and RAW264.7 cells (Figure 2E, F). To determine whether this interaction was direct, GST-pull-down and surface plasmon resonance were performed and showed that GST-fused TRPV2 could readily pull down His - tagged S-RBD protein (Figure 2G), with an equilibrium dissociation constant of 133.4 nM (Figure 2H). Moreover, we performed an indirect IF assay to examine the subcellular localization of TRPV2 and S-RBD in RAW264.7 cells. The RAW264.7 cells were cultured *in vitro* in the presence of His - S-RBD protein (0.5 μg/mL) and His - Vector protein (0.5 μg/mL) at 37 °C or 39.5 °C for 24 h. After washing with PBS, the cells were incubated with anti - TRPV2, anti - S-RBD, and anti - His antibodies. Confocal microscopy images showed that both TRPV2 and S-RBD were predominantly localized in the cell membrane and cytoplasm, with similar distribution patterns at 39.5 °C with a higher overlap ratio than at 37 °C (Figure 2I, Figure S1A). These results verify the interaction between TRPV2 and S-RBD protein at 39.5 °C, which may mediate virus infection in macrophages.

### Febrile temperature promotes cytokine secretion by SARS-CoV-2 S infection of PBAMs

**Figure 3:**
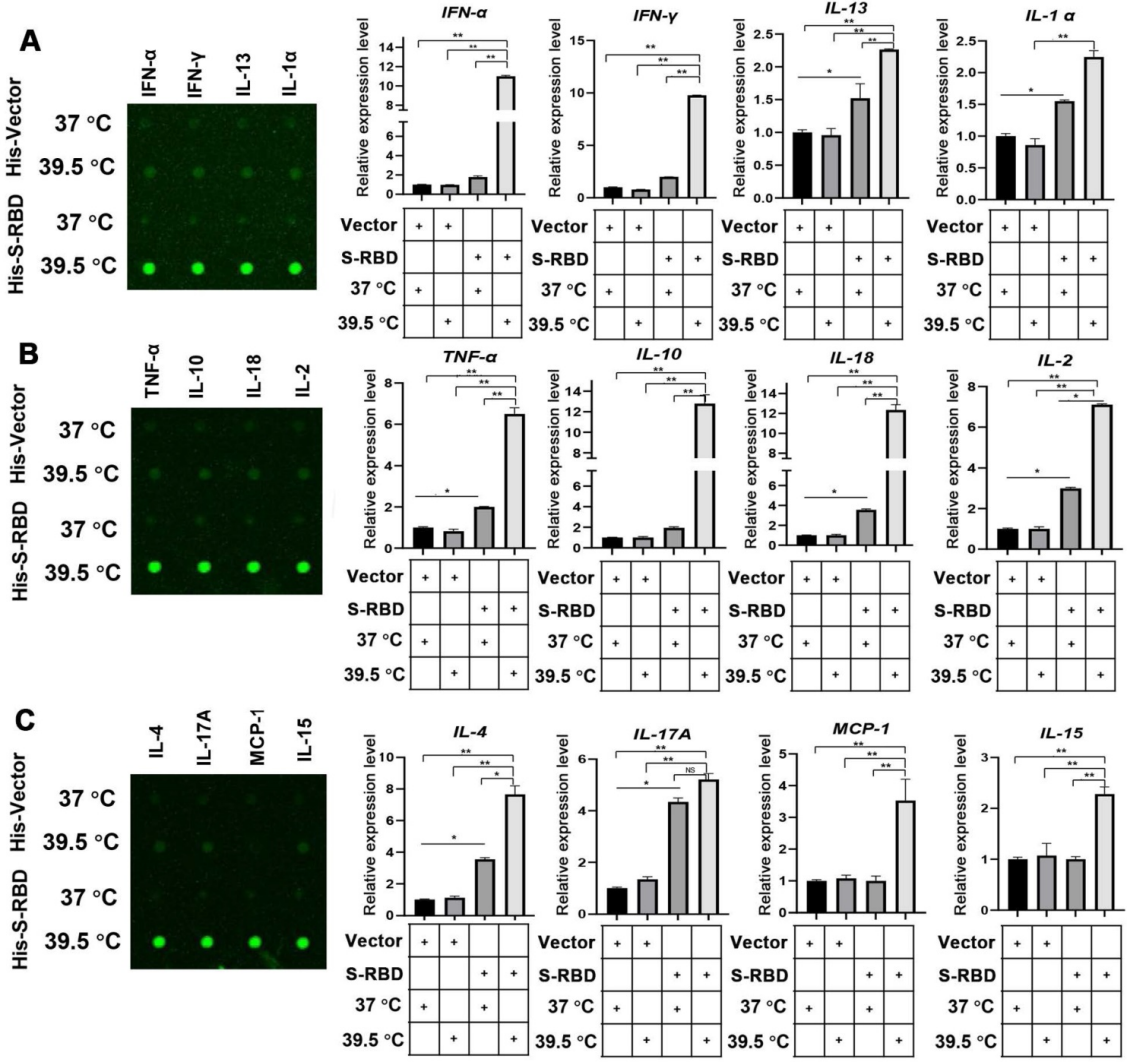
** Cytokine expression level changes in PBAMs following SARS-CoV-2 S treatment in the presence of febrile temperature. (A-C)** Cytokine array analysis showing significant changes in the expression of multiple proteins. Four cytokines, IFN-α (p < 0.01), IFN-γ (p < 0.01), IL-13 (p < 0.01), IL-α (p < 0.01), TNF-α (p < 0.01), IL-10 (p < 0.01), IL-18 (p < 0.01), IL-2 (p < 0.01), IL-4 (p < 0.01), IL-17A (p < 0.01), MCP-1 (p < 0.01), and IL-15 (p < 0.01) exhibited robust increases in the His - S-RBD treatment group at 39.5 °C compared to the control group. Data are shown as the mean ± SD (n = 3). *p < 0.05. **p < 0.01. NS: not significant.

To understand the role of fever in the macrophage response and related innate immunity, we performed bovine cytokine array analysis to identify changes in cytokine expression levels following SARS-CoV-2 S treatment. His - S-RBD protein (0.5 μg/mL) or His - Vector (0.5 μg/mL) were incubated with bovine alveolar macrophages, and the cell culture supernatants were collected after 24 h at 37 °C or 39.5 °C. With respect to the 30 cytokines examined, the febrile temperature (39.5 °C) did not affect the His - Vector treatment groups but robustly enhanced the secretion of 12 cytokines in the His - S-RBD treatment groups. The febrile temperature (39.5 °C) significantly enhanced the expression of macrophage-secreted cytokines, including IFN-α (p < 0.01), IFN-γ (p < 0.01), IL-13 (p < 0.01), IL-α (p < 0.01), TNF-α (p < 0.01), IL-10 (p < 0.01), IL-18 (p < 0.01), IL-2 (p < 0.01), IL-4 (p < 0.01), IL-17A (p < 0.01), MCP-1 (p < 0.01), and IL-15 (p < 0.01) (Figure 3A-C, Table S). However, the cytokine levels of IL-1 F5, IL-21, IP-10, and MIG were not significantly affected (Figure S2). Previous studies showed that the process of SARS-CoV-2 entering human cells is mediated by ACE2 and Neuropilin-1, which act as cell membrane receptors that bind to S on the surface of SARS-CoV-2 11, 12. To understand whether the mechanism underlying the SARS-CoV-2 S-mediated increase in cytokine secretion depends on ACE2 or Neuropilin-1 at 39.5 °C, we constructed knockdown shRNAs for ACE2 and Neuropilin-1 proteins, respectively. After transfecting the PBAMs, we detected the expression of INF-α and IL-13. The results showed that S-RBD increased the release of inflammatory factors at 39.5 °C independently of ACE2 and Neuropilin-1 (Figure S3).

### SARS-CoV-2 S promotes cytokine secretion by interacting with TRPV2 in PBAMs

**Figure 4:**
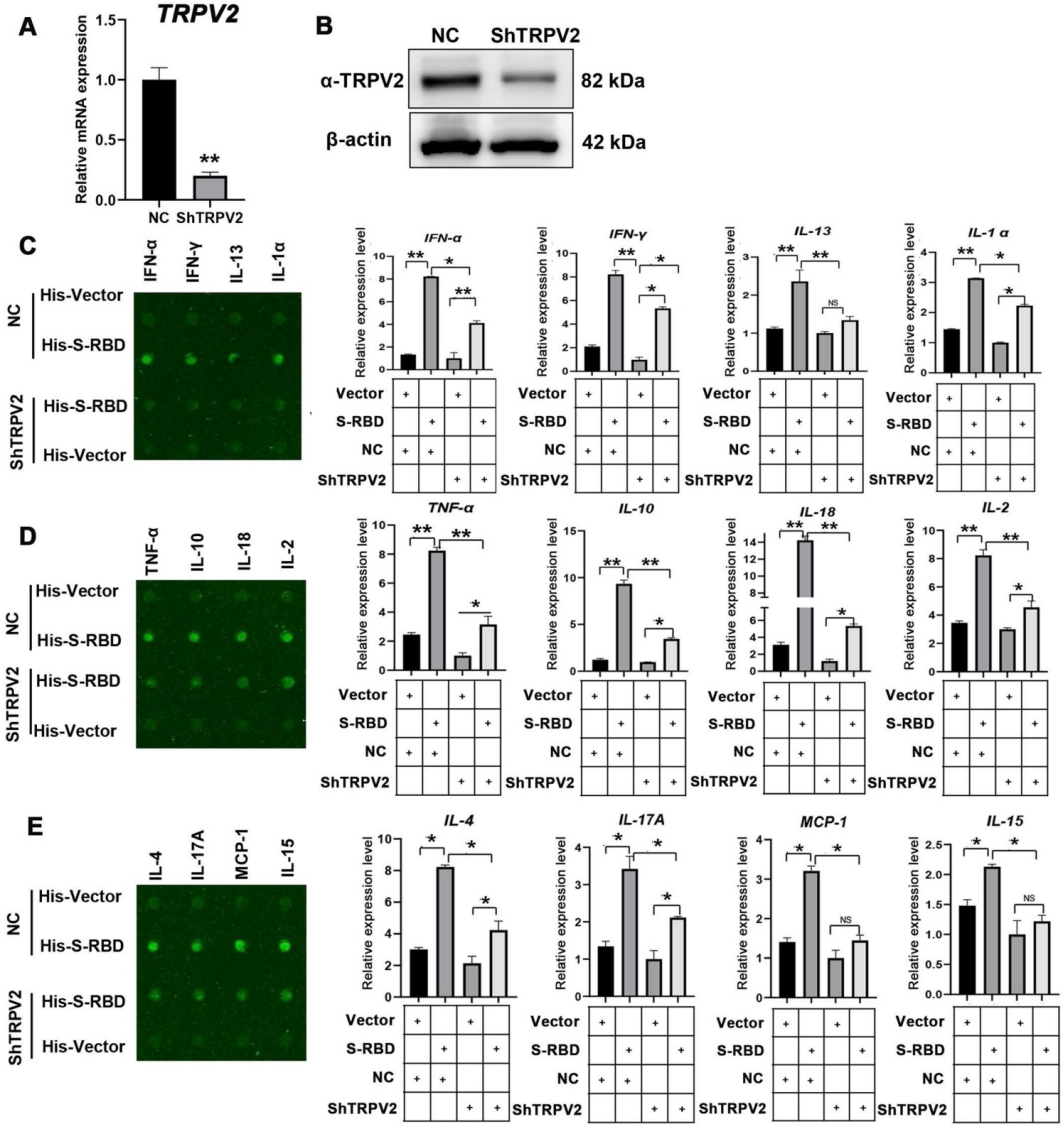
** Cytokine expression level changes with knockdown of TRPV2 expression in PBAMs following SARS-CoV-2 S treatment in the presence of febrile temperature. (A, B)** Depletion of TRPV2 in PBAM using specific shRNAs was performed via lenti-virus transfection. qRT-PCR (A) and western blot assays (B) confirmed TRPV2 knockdown in macrophages. Data are shown as the mean ± SD (n = 3). **p < 0.01. **(C-E)** Cytokine array analysis showing significant changes in the expression of multiple proteins. Twelve cytokines, including IFN-α (p < 0.05), IFN-γ (p < 0.05), IL-13 (p < 0.01), IL-α (p < 0.05), TNF-α (p < 0.01), IL-10 (p < 0.01), IL-18 (p < 0.01), IL-2 (p < 0.01), IL-4 (p < 0.05), IL-17A (p < 0.05), MCP-1 (p < 0.05), and IL-15 (p < 0.05) exhibited robust decreases in His - S-RBD-ShTRPV2 macrophages vs His - S-RBD-NC macrophages at 39.5 °C. Data are shown as the mean ± SD (n = 3). *p < 0.05. **p < 0.01. NS: not significant.

To understand the mechanism underlying the SARS-CoV-2 S-mediated increase in cytokine secretion dependent on TRPV2, we depleted TRPV2 in alveolar macrophages by using shRNAs specifically targeting TRPV2, resulting in significantly decreased TRPV2 mRNA and protein levels (Figure 4A, B). We next performed bovine cytokine array analysis to identify changes in protein expression levels following His - S-RBD (0.5 μg/mL) or His - Vector (0.5 μg/mL) treatment of negative control and ShTRPV2 macrophages at 39.5 °C for 24 h. Interestingly, depletion of TRPV2 in macrophages abolished the SARS-CoV-2 S-promoted secretion of cytokines, including IFN-α (p < 0.01), IFN-γ (p < 0.01), IL-13 (p < 0.01), IL-α (p < 0.01), TNF-α (p < 0.01), IL-10 (p < 0.01), IL-18 (p < 0.01), IL-2 (p < 0.01), IL-4 (p < 0.01), IL-17A (p < 0.01), MCP-1 (p < 0.01), and IL-15 (p < 0.01) (Figure 4C-E). However, the cytokine levels of IL-1 F5, IL-21, IP-10, and MIG were not significantly affected (Figure S4).

### TRPV2 inhibitor SKF-96365 inhibits cytokine secretion in PBAMs induced by SARS-CoV-2 S treatment

**Figure 5:**
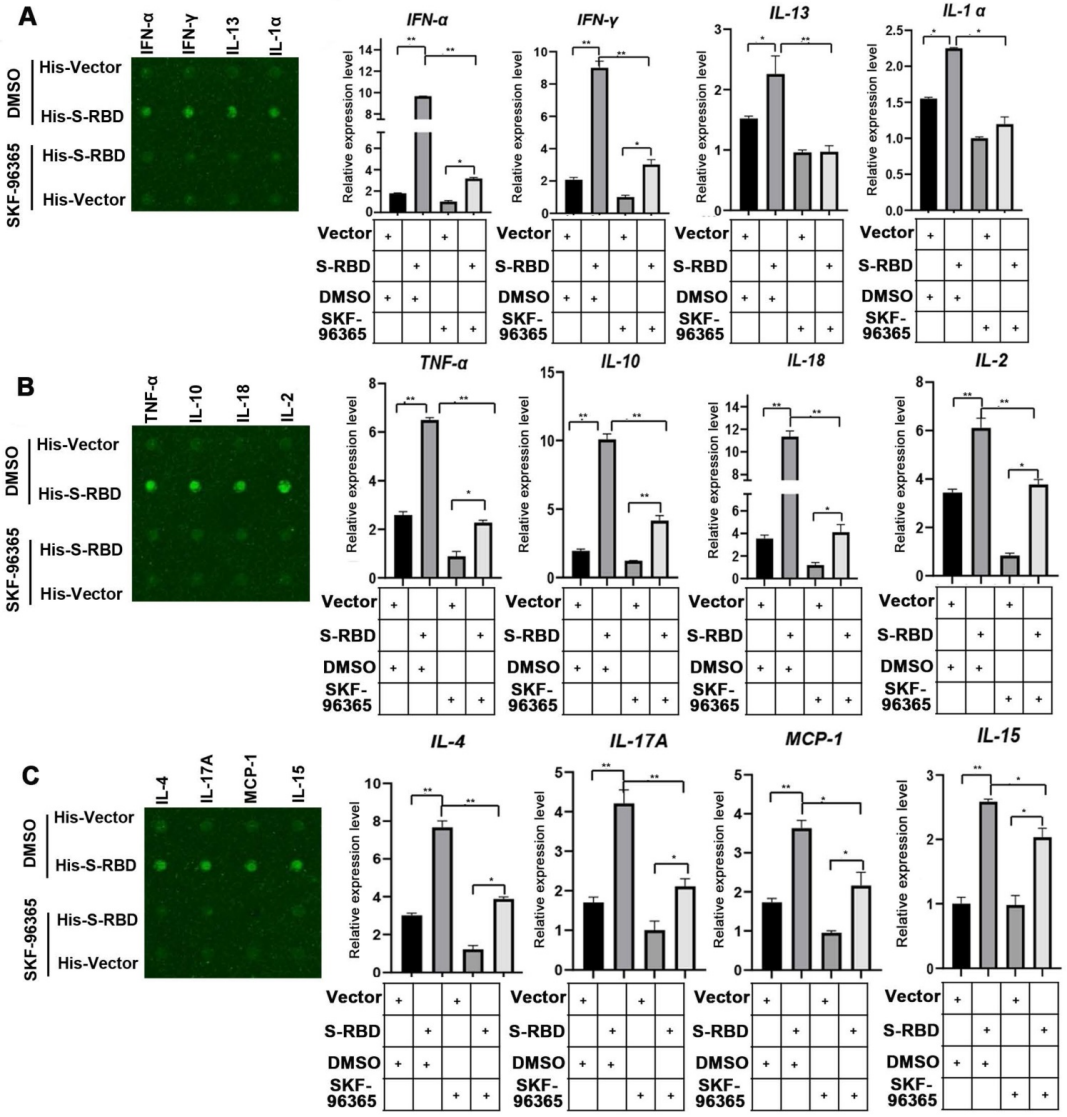
** Cytokine expression level changes in PBAMs treated with SKF-96365 following SARS-CoV-2 S treatment under febrile temperature. (A-C)** Cytokine array analysis showing significant changes in the expression of multiple proteins. Twelve cytokines, including IFN-α (p < 0.01), IFN-γ (p < 0.01), IL-13 (p < 0.01), IL-α (p < 0.05), TNF-α (p < 0.01), IL-10 (p < 0.01), IL-18 (p < 0.01), IL-2 (p < 0.01), IL-4 (p < 0.01), IL-17A (p < 0.01), MCP-1 (p < 0.05), and IL-15 (p < 0.05) exhibited robustly decreased expression in the His - S-RBD-SKF-96365 group (10 µM) vs. His - S-RBD-DMSO group at 39.5 °C. Data are shown as the mean ± SD (n = 3). *p < 0.05.**p < 0.01. NS: not significant.

We then tested whether an inhibitor of TRPV2 (SKF-96365) also decreased cytokine secretion in PBAMs. The macrophages were incubated with His - S-RBD (0.5 μg/mL) or His - Vector (0.5 μg/mL) at 39.5 °C for 24 h, and then the inhibitor SKF-96365 was added to the cultures, which were then incubated for an additional 12 h. The results of the bovine cytokine array were consistent with those of the array performed with TRPV2-knockdown macrophages, showing that SKF-96365 treatment of macrophages abolished the SARS-CoV-2 S-promoted secretion of cytokines, including IFN-α (p < 0.01), IFN-γ (p < 0.01), IL-13 (p < 0.01), IL-α (p < 0.05), TNF-α (p < 0.01), IL-10 (p < 0.01), IL-18 (p < 0.01), IL-2 (p < 0.01), IL-4 (p < 0.01), IL-17A (p < 0.01), MCP-1 (p < 0.05), and IL-15 (p < 0.05) (Figure 5A-C, Table S). However, the cytokine levels of IL-1 F5, IL-21, IP-10, and MIG were not significantly affected (Figure S5). Collectively, these observations indicate that SKF-96365 effectively suppresses the cytokine storm caused by SARS-CoV-2 in the presence of fever, thereby playing a role in the treatment of COVID-19.

### Ca ^2+^-NF-κB pathway is involved in the enhanced cytokine secretion after SARS-CoV2-S stimulation at the temperature of 39.5 °C

**Figure 6:**
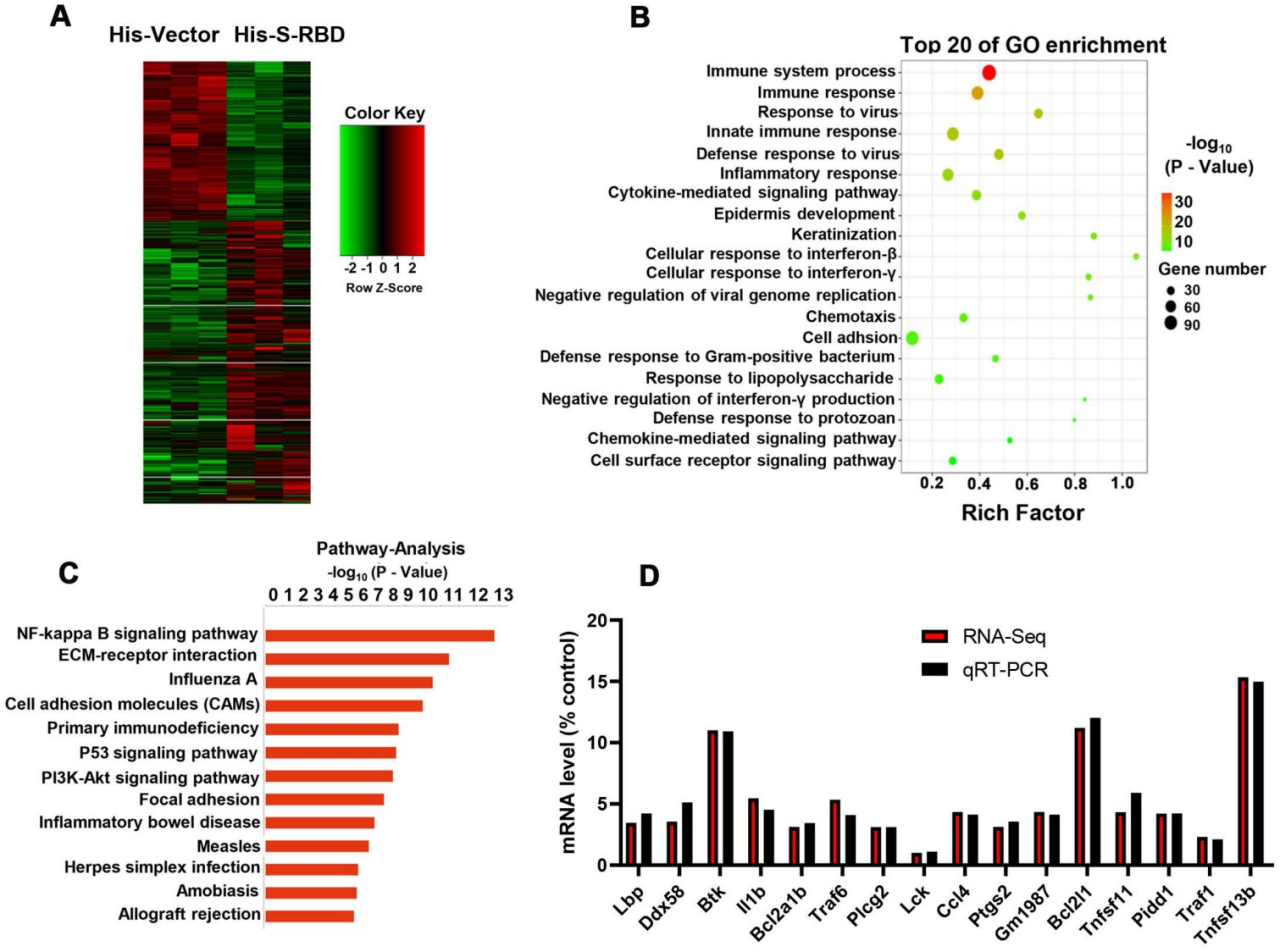
** SARS-CoV-2 S treatment at 39.5 °C to promote cytokine secretion depends on the NF-κB pathway. (A)** Schematic of sample preparation for RNA-seq experiment. **(B, C)** Top 10 GO terms (B) and pathway (C) enriched in differentially expressed genes are shown in (A). **(D)** Comparison of gene expression values obtained by RNA-seq and qRT-PCR. Data are shown as the mean ± SD (n = 3).

**Figure 7:**
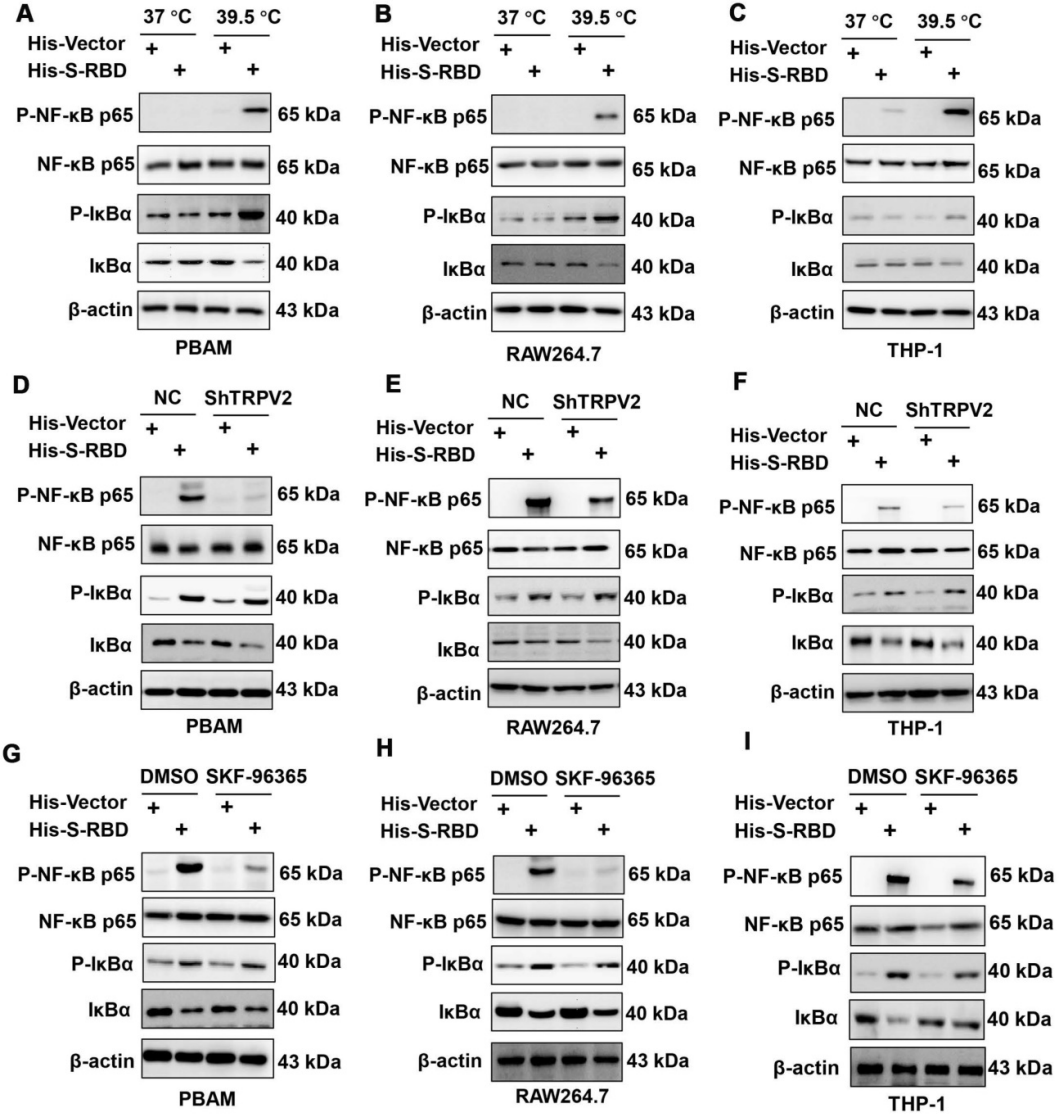
** SARS-CoV-2 S treatment of macrophages at 39.5 °C can activate the NF-κB signaling pathway. (A-C)** Western blotting analysis showed that p-NF-κB p65 and p**-**IκBα expression was increased following stimulation of SARS-CoV2 S-RBD in PBAMs (A), RAW264.7 cells (B), and THP-1 cells (C). **(D-F)** Western blotting analysis showed that p-NF-κB p65 and p**-**IκBα expression was decreased in the ShTRPV2 group compared with the control groups in PBAMs (D), RAW264.7 cells (E), and THP-1 cells (F), respectively. **(G-I)** Western blotting analysis showed that p-NF-κB p65 and p**-**IκBα expression was decreased in the SKF-96365 group compared with control groups in PBAMs (G), RAW264.7 cells (H), and THP-1 cells (I), respectively.

To examine the downstream mechanisms that enhance cytokine secretion, we collected PBAMs with His - S (0.5 μg/mL) or His - Vector (0.5 μg/mL) at 39.5 °C for RNA sequencing (RNA-seq) analysis (Figure 6A). Gene ontology (GO) and pathway analysis revealed enrichment of many genes related to NF-κB signaling, immune responses, and cytokine signaling (Figure 6B, C). We then performed real-time PCR (RT-PCR) to validate the results of RNA-seq analysis using the same RNAs from the RNA-seq analysis. All genes downstream of NF-κB signaling were upregulated, with similar changes observed by RT-PCR and RNA-seq (Figure 6D). Furthermore, we examined the phosphorylation(p)-NF-κB p65, NF-κB p65, p-IκBα, and IκBα by western blotting of PBAMs expressing SARS-CoV-S for 24 h at 39.5 °C or 37 °C. Expression of SARS-CoV2-S in PBAMs for 24 h at 39.5 °C resulted in increased p-NF-κB p65 and p-IκBα as compared to the levels observed at 37 °C-with SARS-CoV2-S or His - Vectors (Figure 7A). The same results were observed in RAW264.7 and THP-1 cells (Figure 7B,C). Moreover, we depleted TRPV2 in PBAMs using TRPV2-specific shRNA (Figure 4A, B) and observed a significant decrease in p-NF-κB p65 and p-IκBα as compared to in the NC group when the cells were incubated with SARS-CoV2-S at 39.5 °C (Figures 7D). The same phenomenon was observed in RAW264.7 and THP-1 cells (Figures 7E, F). Furthermore, we performed western blotting to explore the inhibitory effect of SKF-96365 on p-NF-κB p65, NF-κB p65, p-IκBα, and IκBα. The results showed a significant decrease in p-NF-κB p65 and p-IκBα as compared to in the dimethyl sulfoxide (DMSO)-treated group when incubated with SARS-CoV2-S at 39.5 °C (Figure 7G). The same inhibition by SKF-96365 was also observed in RAW264.7 and THP-1 cells (Figure 7H, I).

**Figure 8:**
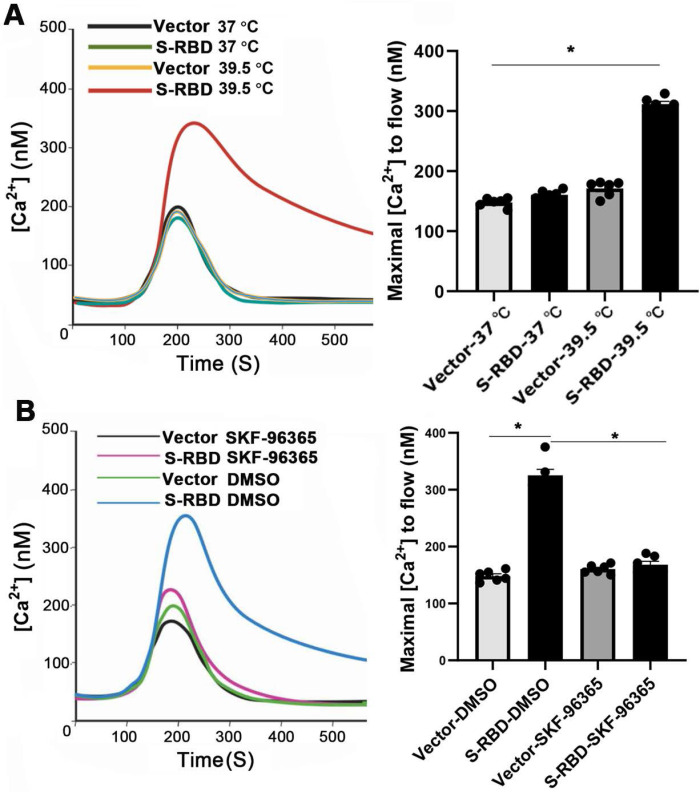
** SARS-CoV-2 S-RBD treatment of PBAMs at 39.5 °C can activate Ca ^2+^ influx. (A)** Kinetic curves (left) and mean data (right) demonstrating that S-RBD enhances Ca ^2+^ channel at 39.5 °C in PBAMs. Data are expressed as the mean ± SEM (n = 6), *p < 0.05. **(B)** Kinetic curves (left) and mean data (right) demonstrating that SKF-96365 decrease Ca ^2+^ channel at 39.5 °C in PBAMs. Data are expressed as the mean ± SEM (n = 6), *p < 0.05.

TRPV2 channels are known to be activated by heating, which increases intracellular Ca ^2+^ levels at 50 °C. We further examined the functional responses of PMABs in terms of Ca ^2+^ following stimulation with His - S-RBD (0.5 μg/mL) or His - Vector (0.5 μg/mL) at 37 °C and 39.5 °C. The results showed that S-RBD can significantly increase the intracellular Ca ^2+^ level of PBAMs at 39.5 °C but the change was minimal at 37 °C (Figure 8A). Furthermore, SKF-96365-treated cells intracellular Ca ^2+^ level lower than DMSO-treated cells in the presence of His - S-RBD (0.5 μg/mL) at 39.5 °C (Figure 8B).

Here, we determined a novel mechanism of SARS-CoV2 infection of macrophages and showed that SKF-96365 is potential candidate for treating SARS-CoV2 in febrile conditions.

## Discussion

Our results clearly show that febrile temperatures promote cytokine storm in macrophages caused by SARS-CoV-2 S infection. Mechanistically, SARS-CoV-2 S-RBD specifically interacts with TRPV2 to activate Ca ^2+^ entry, leading to NF-κB p65 phosphorylation, thereby enhancing the secretion of cytokines (TNF-α and others). SKF-96365 effectively repressed cytokine secretion promoted by SARS-CoV-2 S-RBD in PBAMs.

Emerging human CoVs that have appeared in the past two decades, such as SARS, Middle East respiratory syndrome CoV, and SARS-CoV-2, all have a broad host range with great potential to infect multiple species 3, 4, 13. SARS and Middle East respiratory syndrome possibly originated from bats and were transmitted from intermediate animal hosts, civet cats, and camels, respectively, to humans 14, 15. A recent study showed that the genome sequence of SARS-CoV-2 is 96.2% identical to that of a SARS-like bat virus, bat CoV RaTGB, whereas SARS-CoV-2 shares 79.5% identity with SARS-CoV 16. Thus, bats may be the primary reservoir of this virus based on genomic analysis. Moreover, protein sequence alignment and phylogenetic analysis showed that similar residues in the ACE2 receptor were observed in many species 13, such as turtles, pangolin, and snakes, which are all possible intermediate hosts of SARS-CoV-2. Here, we found that human SARS-CoV-2 S-RBD protein interacts with TRPV2 in bovine alveolar macrophages, RAW264.7 mouse macrophages, and THP-1 human macrophages.

TRPV2 is considered as a noxious heat sensor channel, and TRPV2 channels can open in response to noxious temperatures greater than 52 °C 17. In addition, TRPV2 plays an important role in osmosensory and mechanosensory processes, and this channel can open in response to many other stimuli, such as hormones, growth factors (IGF-1, PDGF), and neuropeptide head activator 5. In *Homo sapiens*, TRPV2 is also expressed in the lymph nodes, spleen, placenta, lungs, and appendix, with the highest expression in the lungs. In contrast, in bovines, TRPV2 is broadly expressed in the thymus, cerebellum, placenta, and spleen, with TRPV2 most commonly expressed in the thymus. The thymus gland is an immune organ with an important role in the immune system and is the site of T cell maturation. Previous studies reported that TRPV2 is a key participant in the earliest stages of macrophage phagocytosis. TRPV2-null mice exhibit accelerated mortality following challenge with pathogenic *Listeria monocytogenes*. This suggests that TRPV2 plays an important role in the adaptive immune system 18.

In summary, our data indicate that febrile temperatures promote cytokine secretion through SARS-CoV-2 S-mediated infection, and that knockdown or inhibition (SKF-96365) of TRPV2 significantly decreases the release of cytokines that drive the inflammatory response. TRPV2 is physically associated with the SARS-CoV-2 S-RBD protein. Therefore, TRPV2 may serve as a therapeutic target for COVID-19.

## Supplementary Material

Supplementary figures.

Supplementary tables.

## Abbreviations

ACE2: angiotensin-converting enzyme 2
BCoV: bovine coronavirus
COVID-19: coronavirus disease 2019
SARS-CoV-2: severe acute respiratory syndrome coronavirus-2
TRPV2: transient receptor potential vanilloid 2
PBAM: primary bovine alveolar macrophage
