# Supplementary material for: TRPV2-spike protein interaction mediates the entry of SARS-CoV-2 into macrophages in febrile conditions
Source: Theranostics. 2021 May 25;11(15):7379–90. doi: 10.7150/thno.58781 (PMC8210595; doi:10.7150/thno.58781)
Supplement: Supplementary file 1 — Supplementary figures. [file thnov11p7379s1.pdf]

Supplementary Figures

TRPV2–spike protein interaction mediates the entry of SARS-CoV-2 into macrophages in febrile conditions

- Jinrui Xu[1,2*], Yuquan Yang[3*], Zhaoyuan Hou[3#], Hao Jia[3#], Yujiong Wang[1,2#]
- [1] Key Laboratory of the Ministry of Education for Conservation and Utilization of Special Biological Resources in the Western, Yinchuan 750021, China
- [2] College of Life Science, Ningxia University, Yinchuan 750021, Ningxia, China
- [3] Faculty of Basic Medicine, Shanghai Jiao tong University School of Medicine, Shanghai, China

- *: These authors contributed equally to this paper
- #: Corresponding authors

Figure.S1

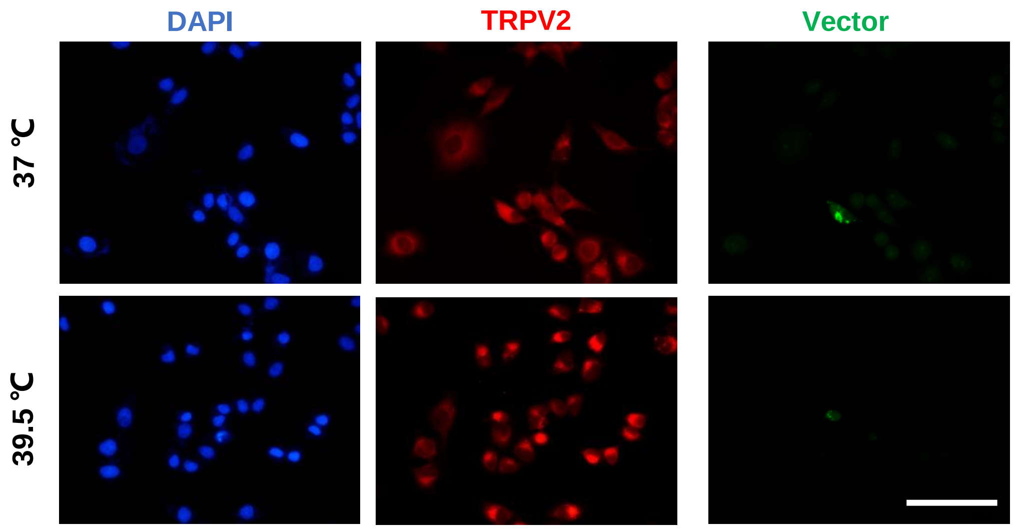

IF performed with primary anti-TRPV2 and anti-His antibodies.

DAPI: nuclear staining. Scale bars represent 20 µm.

Figure.S2

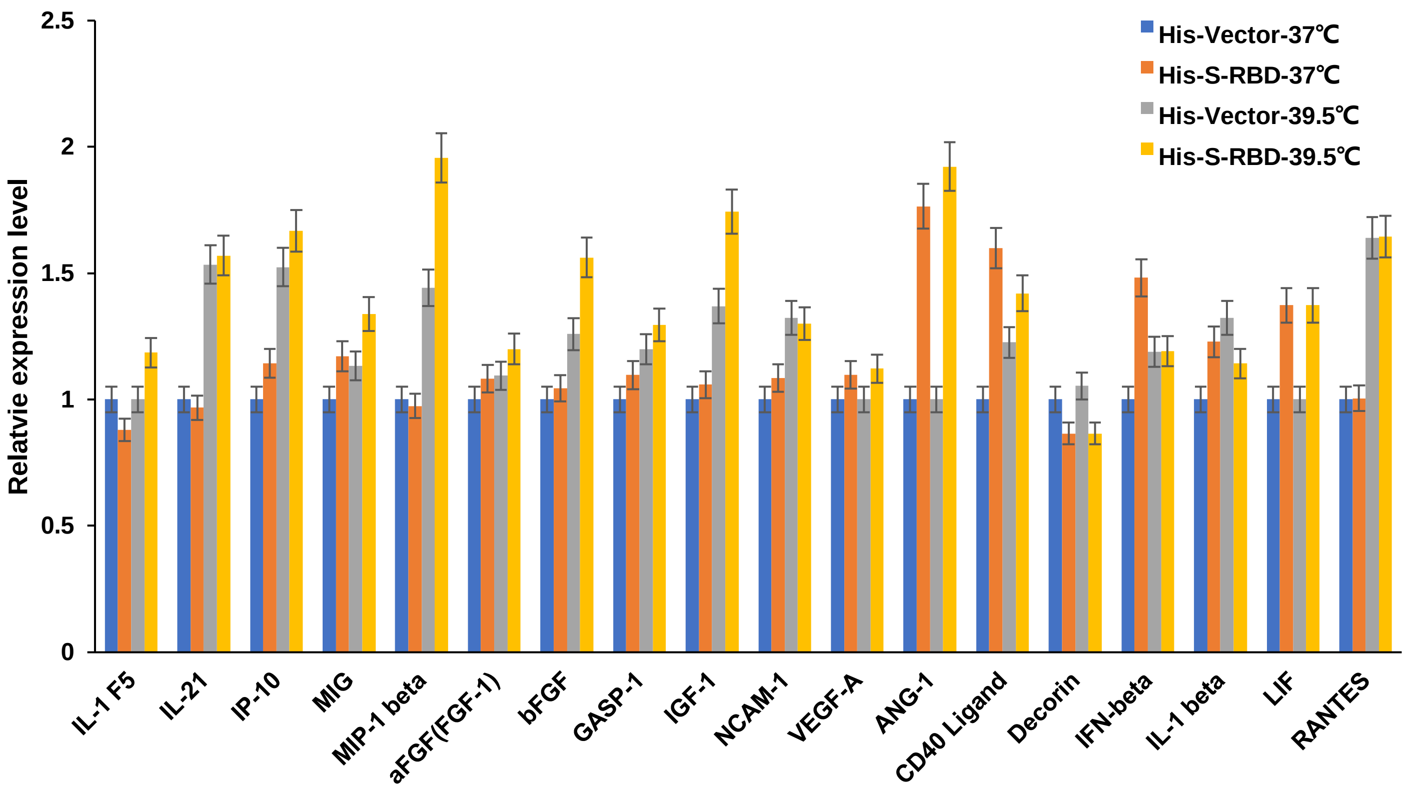

The cytokines amount for IL-1 F5, IL-21, IP-10, MIG, etc. were not significantly affected in His-S-RBD treatment group under 39.5 ℃ vs control group.

Figure.S3

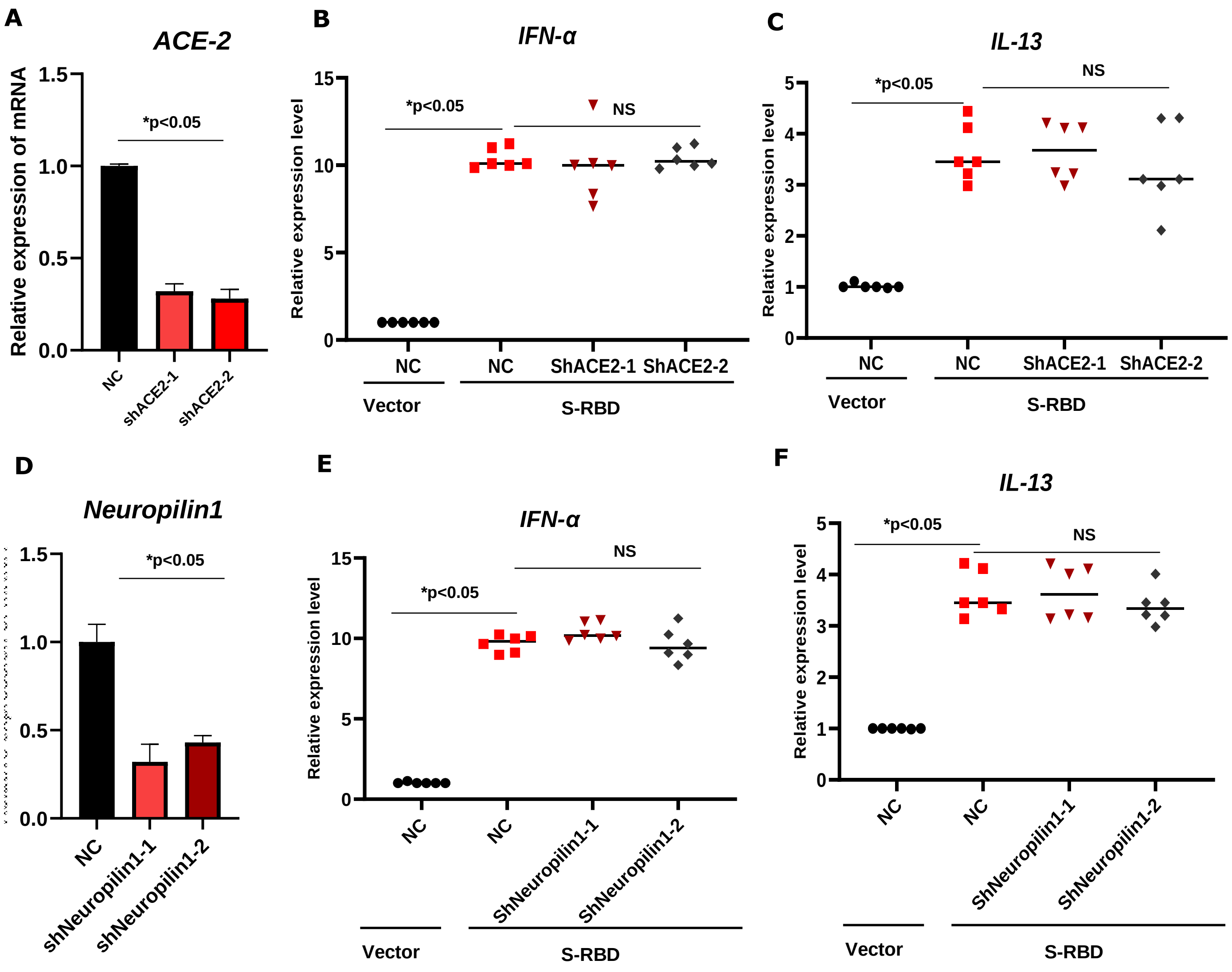

(A) Depletion of ACE-2 in PBAMs using specific shRNAs was performed via lenti-virus transfection. Data are shown as the mean ± SD from three independent experiments. *p < 0.05.
(B) (C) Compared with the control group, the His-S-RBD treatment group has a significant effect on IFN-α(B), IL-13(C) when the temperature is at 39.5 ℃. In the ShACE2-1 and -2 groups, there was no significant difference between the S-RBD treatment and the NC group. Data are shown as the mean ± SD from six independent experiments. *p < 0.05.
(D) Depletion of Neuropilin1 in PBAMs using specific shRNAs was performed via lenti-virus transfection. Data are shown as the mean ± SD from three independent experiments. *p < 0.05. NS: no significant.
(E) (F) Compared with the control group, the His-S-RBD treatment group has a significant effect on IFN-α(E), IL-13(F) when the temperature is at 39.5 ℃. In the ShNeuropilin-1 and -2 groups, there was no significant difference between the S-RBD treatment and the NC group. Data are shown as the mean ± SD from six independent experiments. *p < 0.05.

Figure.S4

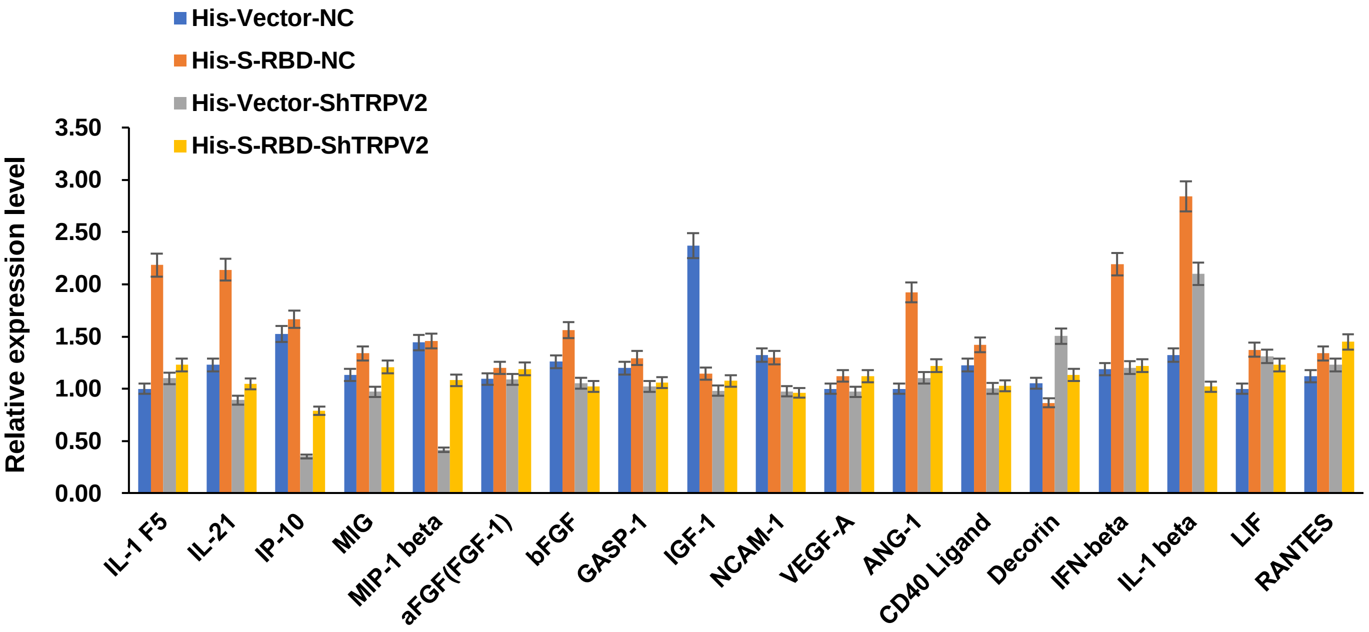

The cytokines amount for IL-1 F5, IL-21, IP-10, MIG, etc. were not significantly affected in His-S-RBD-shTRPV2 vs control group under 39.5 ℃.

Figure.S5

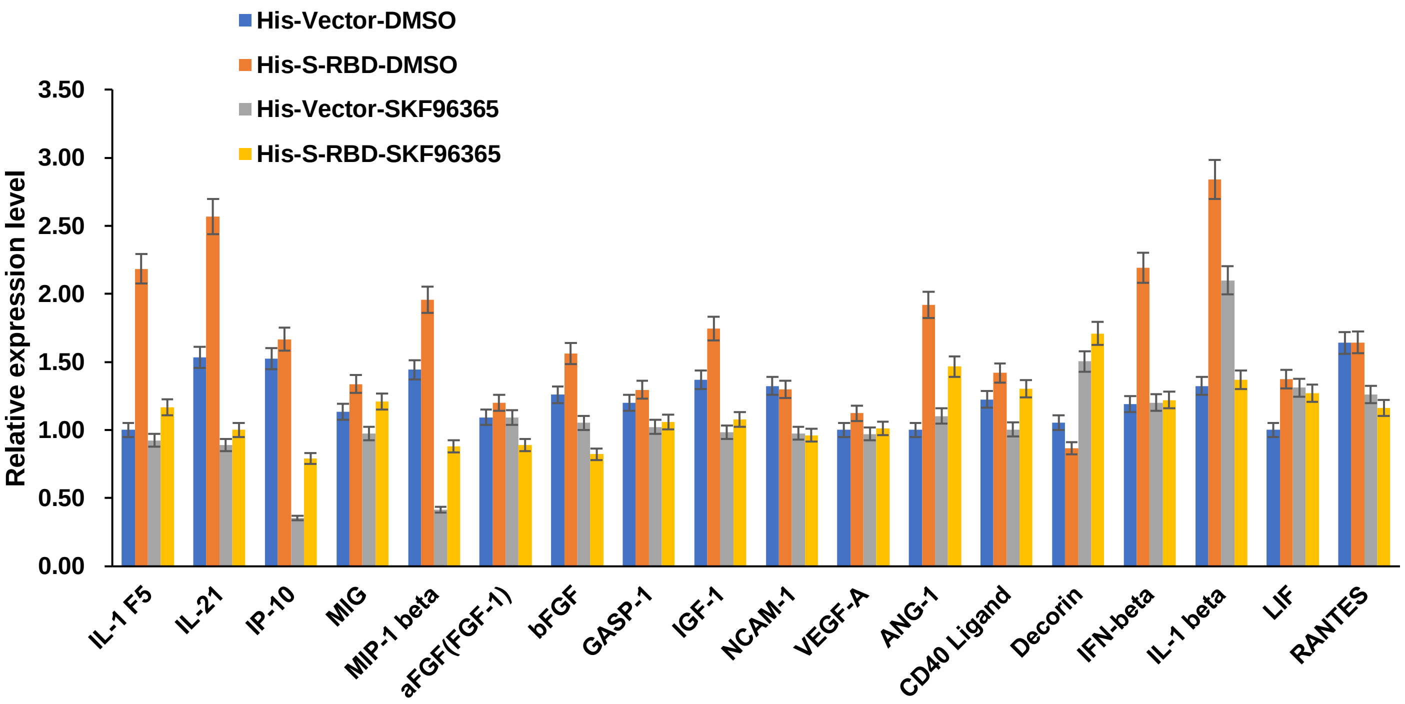

The cytokines amount for IL-1 F5, IL-21, IP-10, MIG, etc. were not significantly affected in His-S-RBD-SKF96365 vs control group under 39.5 ℃.
